# A Retrospective Analysis of the Efficacy of Silicone Sling in the Management of Severe Congenital Ptosis

**DOI:** 10.7759/cureus.77134

**Published:** 2025-01-08

**Authors:** Prolima Thacker, Richa Agarwal, Anju Rastogi, Annu Joon, Himali Agarwal, Pankaj Aggarwal

**Affiliations:** 1 Ophthalmology, Dr. Ram Manohar Lohia Institute of Medical Sciences, Lucknow, IND; 2 Ophthalmology, All India Institute of Medical Sciences, Gorakhpur, Gorakhpur, IND; 3 Ophthalmology, Maulana Azad Medical College, Delhi, IND; 4 Ophthalmology, Guru Nanak Eye Centre, Delhi, IND; 5 Orthopedics, Dr. Ram Manohar Lohia Institute of Medical Sciences, Lucknow, IND

**Keywords:** blepharoptosis, congenital ptosis, frontalis suspension, lid height asymmetry, marginal reflex distance (mrd), silicone band

## Abstract

Introduction: Frontalis sling surgery is indicated in severe ptosis with poor levator action. A number of new sling materials are available, including silicone sling. The efficacy of sling surgery with silicone sling was analyzed.

Method: A retrospective study was conducted where records of patients who underwent frontalis sling surgery using silicone slings between July 2014 and June 2016 in a single center were analyzed. Patients had a follow-up of at least three years. The amount of correction achieved and complications, if any, were noted. Mean preoperative Marginal Reflex Distance 1 (MRD1) and mean postoperative MRD1 values were compared at six months, one year, and three years using a paired "t"* *test. Lid height asymmetry (LHA) was also measured preoperatively and compared with the postoperative values at six months, one year, and three years after surgery using the paired "t"* *test.

Results: About 27 patients underwent this procedure. Bilateral sling surgery was performed in all patients. Seventeen patients had severe simple congenital ptosis, three had monocular elevation deficit (MED), six had Marcus Gunn phenomenon (MGP) with ptosis, and one patient had blepharophimosis epicanthus syndrome (BPES). MRD1 was measured preoperatively to be 0.37 ± 1.72 mm. Follow-up MRD1 was measured at six months, one year, and three years postoperatively and was found to be 4.46 ± 1.28, 4.26 ± 1.46, and 3.79 ± 1.72 mm, respectively. Mean preoperative MRD1 and mean postoperative MRD1 values were compared at six months, one year, and three years using a paired "t"* *test and were found to be statistically significant (p *<* 0.001) at all follow-ups. Lid height asymmetry (LHA) was measured at 1.74 ± 3.96 mm preoperatively. Postoperatively, LHA was measured at six months, one year, and three years and was found to be 0.44 ± 1.18, 0.59 ± 1.3, and 0.89 ± 1.60 mm, respectively. On comparing mean preoperative LHA and mean postoperative LHA values at six months (p = 0.0006), one year (p = 0.0023), and three years (p = 0.0253) using a paired "t"* *test, the difference was found to be statistically significant at all follow-up visits.

At the end of the three-year follow-up, good correction was seen in 48.15% (13/27) of the cases, fair correction was seen in 29.62% (8/27) of the cases, and poor in 22.22% (6/27) of the cases.

A satisfactory correction was seen in 19 patients (70.37%). Complications seen were under-correction in one eye, lid notch in one eye, tube exposure in two eyes, suture granuloma in one eye, and late recurrence of ptosis in four eyes.

Conclusion: Silicone sling surgery is an effective surgery with few complications and a stable outcome even after three years of follow-up.

## Introduction

Congenital ptosis is associated with reduced levator function and a weak or absent lid crease [1]. Correction of the ptosis is indicated for both cosmetic and functional reasons. Surgery is indicated when the ptotic lid obstructs the visual axis, leading to amblyopia, cosmoses, or abnormal head posture.

Frontalis sling surgery is performed when the levator function is poor [2-4]. Apart from simple congenital ptosis, the frontalis sling procedure is also used to correct complicated ptosis due to myogenic or neurogenic causes, such as cranial nerve III palsy, blepharophimosis syndrome, monocular elevation deficit (MED), and Marcus-Gunn jaw winking syndrome.

Many different sling materials are available for frontal sling surgery. Autogenous fascia lata is considered the material of choice with comparably low rates of recurrence [2-6]. Autogenous fascia lata is difficult to harvest, the amount of material harvested may be insufficient, and it is associated with postoperative leg scarring [7,8]. Efforts continue to develop new sling materials to overcome some of these problems. Preserved fascia lata (PFL) and synthetic materials are also commonly used, including silicone rods, polyester, nylon, polytetrafluoroethylene, and polypropylene [2,5,6,9-17]. Silicone is an inert material with excellent elasticity and easy adjustability. It is biocompatible with lower risks of scar formation. The additional elasticity of the silicone makes it a suitable agent for correcting ptosis due to neurologic causes [2,5,6,13,18]. We assessed the long-term efficacy of silicone as a suspension material in patients with severe ptosis.

## Materials and methods

A retrospective study was conducted, where we analyzed the medical records of all patients who had undergone the frontalis sling operation for severe ptosis between July 2014 and April 2016 in the pediatric ophthalmology department of a tertiary eye care center. We included all patients who had a frontalis sling surgery using a silicone sling as a suspension material with a minimum follow-up of three years. The frontalis sling operation was done for severe ptosis when levator function was poor and <4 mm [2]. Patients with a history of re-surgery and inadequate follow-up were excluded. Approval was taken from the Maulana Azad Medical College Institutional Ethics Committee (approval no.: 218/24) and the study was in adherence to the tenets of the Declaration of Helsinki.

**Figure 1:**
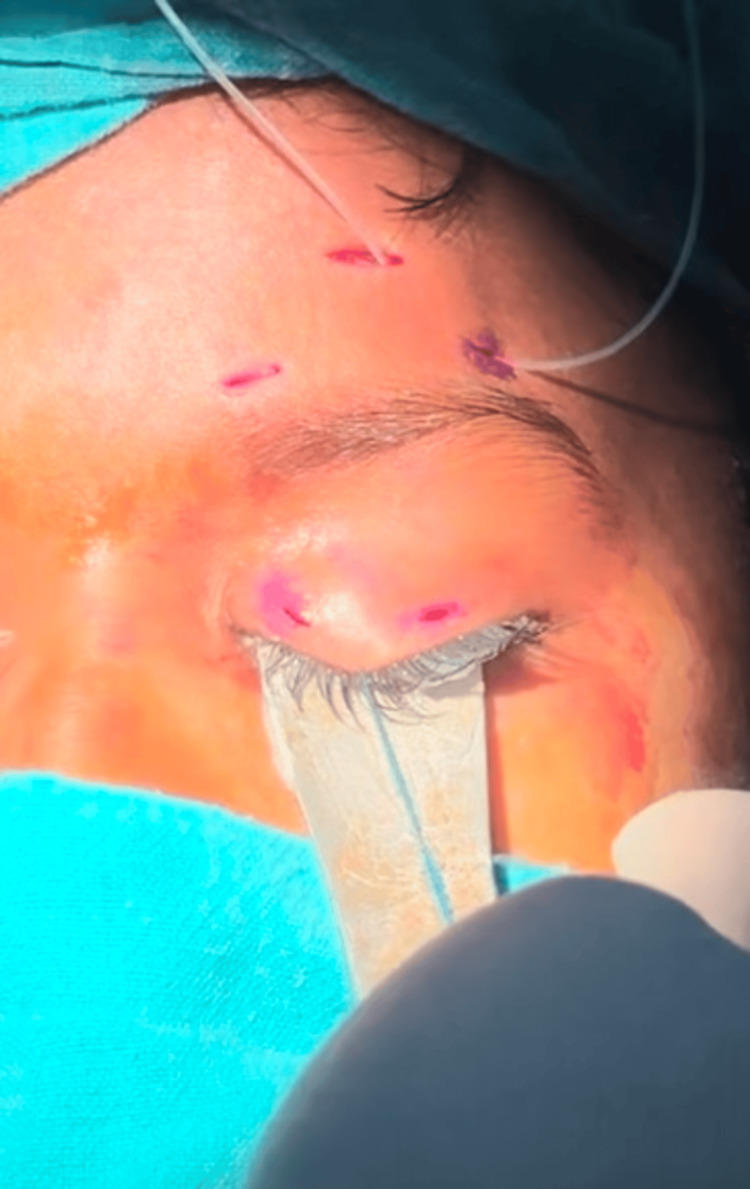
Intraoperative picture showing the passing of the silicone sling in the pentagon Fox's configuration

The silicone sling was passed in a Fox’s pentagonal fashion using the two stab incisions in the lid, two brow incisions, and a forehead incision, down to the periosteum, in line with the center of the pupil (Figure 1).

A pocket was dissected to insert the cut ends of the silicone sling beneath the frontalis muscle in the forehead incision using blunt dissection. The silicone rod was passed from the forehead incision to the brow incision in a pentagonal fashion. When passing the silicone rod through the eyelid incision, a lid plate was placed beneath the lid to protect the globe. This was followed by passing a silicone rod into the sleeve, and then the sleeve was placed into the forehead incision. Then, the eyelid level was adjusted until the eyelid margin was elevated to the superior limbus of the cornea or 1 mm below the superior limbus. The brow and forehead incisions were closed using a 6-0 polyglactin suture followed by skin closure using a 6-0 silk suture. Inverse frost sutures were applied at the end of the surgery to prevent corneal exposure in the immediate postoperative period. Following surgery, lubricating eye drops were applied hourly, and ointments were applied every two hours for one week. Patients were advised to apply the frost sutures at night and when sleeping. The frost sutures were removed at the end of two weeks. The skin sutures were removed at the end of one week.

Clinical outcome and evaluation

Data from follow-up examinations was collected at six months, one year, and three years after surgery. The Marginal Reflex Distance 1 (MRD1), lid height asymmetry (LHA), corneal exposure, and other complications were assessed at each follow-up visit. The postoperative results were considered as good if MRD1 was 3.5 mm or more and asymmetry ≤1 mm; fair if MRD1 was 2.5 to 3.5 mm and asymmetry 1.0 to 2.0 mm; and poor if MRD1 was <2.5 or asymmetry >2.0 mm [18]. Any worsening from good or fair to poor was defined as recurrence.

Statistical analysis

A comparison was done between pre- and postoperative MRD and eyelid height asymmetry using the paired t-test. Statistical analyses were performed using SPSS Statistics for Windows, Version 17 (Released 2005; SPSS Inc., Chicago, United States) and a p-value less than 0.05 was considered statistically significant.

## Results

Between July 2014 and June 2016, 39 patients underwent sling surgery using silicone sling as the suspension material. Of these 39 patients, 27 patients had three years of follow-up and were thus included in the study. Of the total patients analyzed, 17 patients had severe simple congenital ptosis, three had MED, six had Marcus Gunn phenomenon (MGP) with ptosis, and one had blepharophimosis epicanthus syndrome (BPES). The mean follow-up time was 37.8 months. Descriptive statistics for demographic and ophthalmic measurements, including gender, age at surgery, laterality, and preoperative MRD, are described in Table 1.

**Table 1:** Demographics and descriptive statistics for ophthalmic measurement BL: bilateral cases; MRD: margin reflex distance; UL: unilateral cases; mm: millimeters

Demographics	(n = 27)
Gender (male: female)	16:11
Age at operation (years)	13.82 ± 8.24 (3-25 years)
Laterality (bilateral: unilateral)	18:9 (total 45 eyelids)
Preoperative MRD (mm)	(BL) 0.38 ± 0.89
	(UL) 0.33 ± 0.79
	(Overall) 0.37 ± 0.86 mm
Preoperative lid height asymmetry (mm)	1.74 ± 1.97

Associated ophthalmic findings are described in Table 2.

**Table 2:** Associated ocular findings BPES: blepharophimosis epicanthus syndrome

Associated ocular findings	(n = 27)
Amblyopia	6
Strabismus (horizontal)	5
Anisometropia	4
Monocular elevation deficit	3
Marcus gunn phenomenon	6
BPES	1

Amblyopia was present in six patients. Five patients had associated horizontal strabismus. The MGP was present in six patients. Bilateral sling surgery was preceded by a levator excision in these patients to tackle the MGP. Anisometropia was present in four patients. Complicated ptosis with MED was present in three patients. Inferior rectus recession alone or along with Knapp’s procedure was done in these patients to correct the primary position hypotropia. Bilateral sling patients were done in these patients only after ensuring a fair to good Bell’s phenomenon.

MRD1 was measured preoperatively to be 0.37 ± 0.86 mm. Follow-up MRD1 was measured at six months, one year, and three years postoperatively and was found to be 4.46 ± 0.64, 4.26 ± 0.73, and 3.79 ± 0.86 mm, respectively (Table 3).

**Table 3:** Margin reflex distance and eyelid height asymmetry at six months, one year, and three years mm: millimeters; SD: standard deviation

Marginal reflex distance (mm)	Mean ± SD
Six months	4.46 ± 0.64
One year	4.26 ± 0.73
Three years	3.79 ±0.86
Eyelid height asymmetry (mm)	
Six months	0.44 ± 0.59
One year	0.59 ± 0.65
Three years	0.89 ± 0.80

Mean preoperative MRD1 and mean postoperative MRD1 values at six months, one year, and three years were compared using a paired “t” test and were found to be statistically significant (p ≤ 0.0001) at all follow-ups (Table 4).

**Table 4:** Preoperative and postoperative MRD and LHA values with their statistical significance mm: millimeters; MRD: marginal reflex distance; LHA: lid height asymmetry

	Preoperative	Postoperative six months	Postoperative one year	Postoperative three year
Marginal reflex distance (mm) MRD	0.37 ± 0.86	4.46 ± 0.64 (p < 0.0001)	4.26 ± 0.73 (p < 0.0001)	3.79 ± 0.86 (p < 0.0001)
Lid height asymmetry (mm) LHA	1.74 ± 1.97	0.44 ± 0.59 (p = 0.0006)	0.59 ± 0.65 (p = 0.0023)	0.89 ± 0.80 (p = 0.0253)

Results were also compared between mean MRD1 values at six months and one year (p = 0.71) and showed no significant difference in the values. However, comparing the MRD1 value at six months and three years showed a significant difference (p = 0.0009). At the end of the three-year follow-up, four cases had a late recurrence of ptosis, where the patients needed to get re-surgery (Table 3).

LHA was measured at 1.74 ± 1.97 mm preoperatively. Postoperatively, LHA was measured at six months, one year, and three years and was found to be 0.44 ± 0.59, 0.59 ± 0.65, and 0.89 ± 0.80 mm, respectively (Table 3). Mean preoperative LHA and mean postoperative LHA values at six months (p = 0.0006), one year (p = 0.0023), and three years (p = 0.0253) were analyzed using a paired “t” test and were found to be statistically significant at all follow-ups.

**Figure 2:**
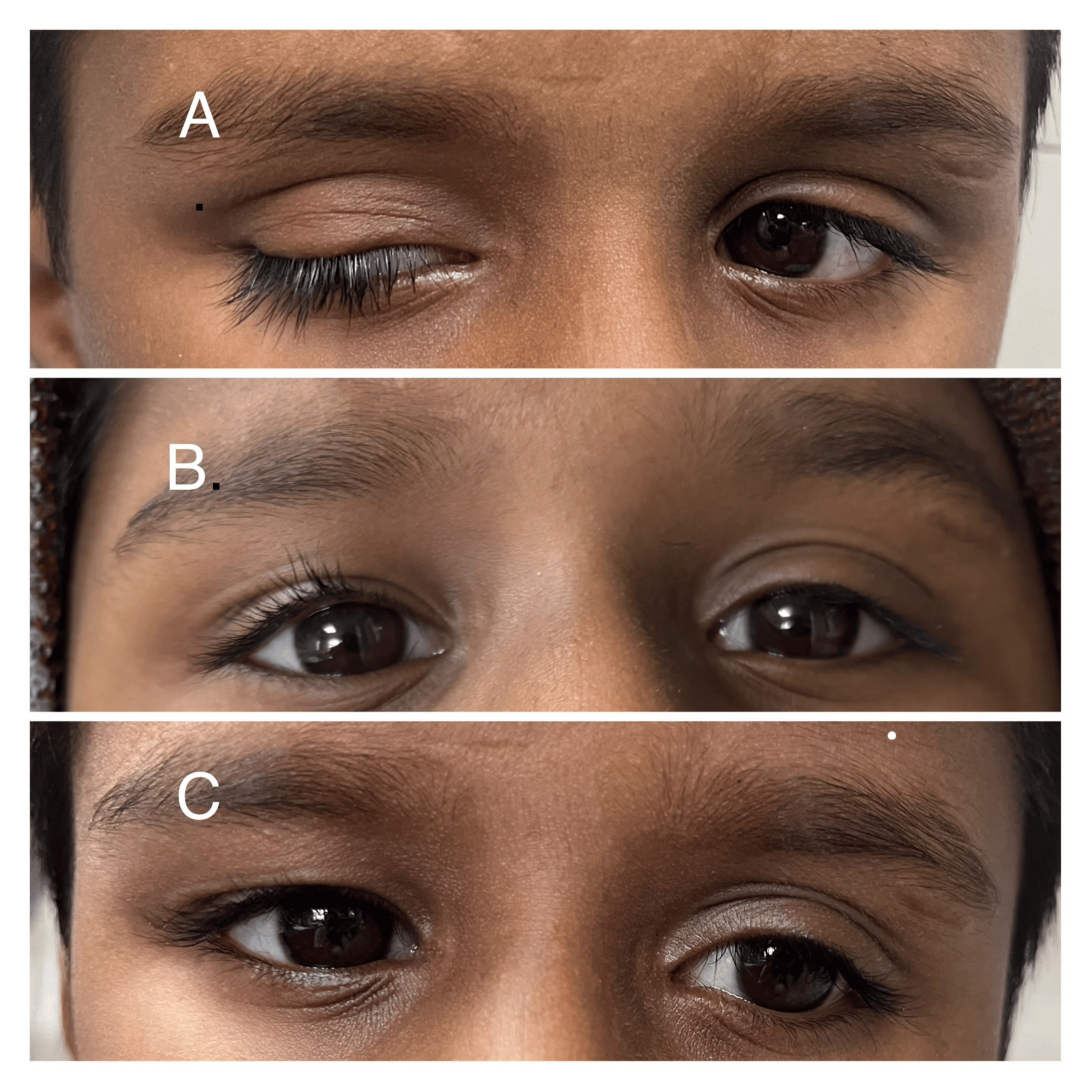
A) Preoperative picture; B) picture at one-year follow-up; C) picture at three-year follow-up

**Figure 3:**
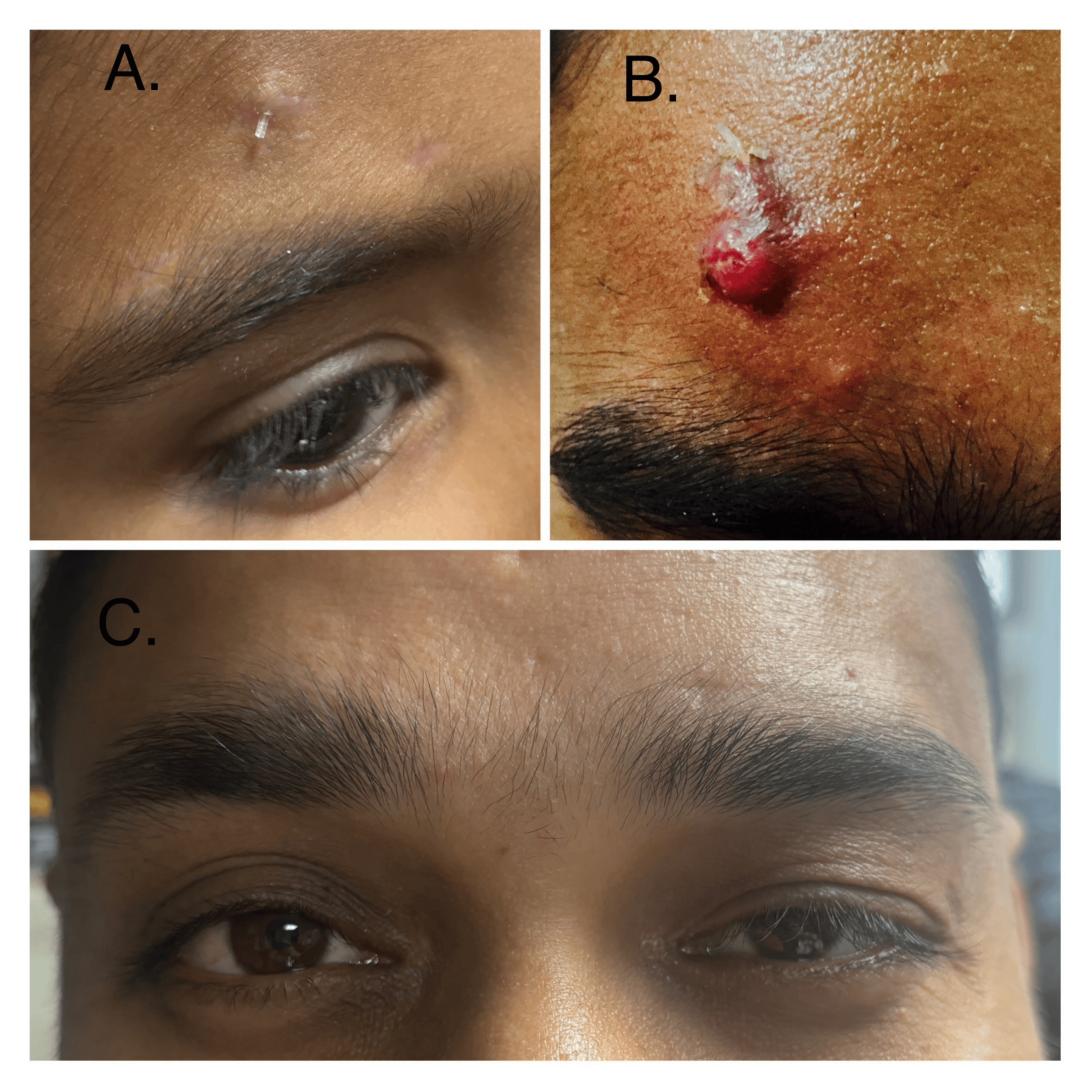
Complications seen in the study: A) tube exposure; B) infective suture granuloma with tube exposure; C) late recurrence

At the end of the three-year follow-up, good correction was seen in 48.15% of the cases, fair correction was seen in 29.62% of the cases, and poor in 22.22% of the cases (Figures 2-3).

Satisfactory postoperative eyelid elevation was defined as an MRD change or elevation ≥2 mm from preoperative MRD1 with an eyelid height asymmetry of <2 mm with an absence of any complications [18]. A satisfactory correction was seen in 19 patients (70.37%). Postoperatively, minimal lagophthalmos was found in 13% of cases at the six-month follow-up. This improved by one year, and no patients had significant lagophthalmos at the end of the one- and three-year follow-ups. The lagophthalmos was managed by frequent instillation of lubricating eye drops to prevent the risk of exposure to keratopathy. No corneal complications were seen in our study.

Silicone rod extrusion and recurrence occurred in one case, and in another case, suture granuloma with tube exposure was seen (Figure 4). The suture granuloma got infected and was managed with systemic antibiotics. The silicone implant had to be removed, and a re-surgery with fascia lata had to be done in this case. Recurrence was seen in four (14.8%) cases.

A satisfactory correction was seen in 19 patients (70.37%). Complications seen were under-correction in two eyes, lid notch in one eye, tube exposure in two eyes, suture granuloma in one eye, and late recurrence of ptosis in four eyes (Table 5).

**Table 5:** Complications seen with silicone sling

Postoperative complications	No. of patients (%) (n = 27)
Under correction	2 (7.4%)
Lid notch	1 (3.7%)
Tube exposure	2 (7.4%)
Suture granuloma	1 (3.7%)
Late recurrence	4 (14.8%)

## Discussion

The results of the study showed that using silicone rods as a sling material provides good cosmesis with few complications and thus is an effective alternative method to fascia lata. The frontalis sling procedure for ptosis correction using a silicone sling was first described by Tillett and Tillett [19]. Other studies have described the use of silicone rods for sling reconstruction with good results [12,19,20,21]. The silicone rod is readily available, easily adjusted, and elastic. These characteristics make the silicone rod a suitable suspensory material for ptosis. We studied the long-term stability of the silicone sling in patients needing sling surgery for severe ptosis.

Our study shows that silicone rod is an effective material for frontalis sling surgery. A satisfactory outcome was seen in 70.37% of our patients. This agrees well with the results of other studies as well [12,22-26]. The correction acquired with silicone sling persisted through three years, with the p-value remaining statistically significant between the preoperative MRD1 and postoperative MRD1 at the three-year follow-up. However, due to the late recurrence seen in four patients, the difference in the preoperative MRD1 and the postoperative MRD1 at three years lost its statistical significance. We can postulate that there is a slight attrition of the correction in MRD1 with time when using a silicone sling. Despite this, 70.37% of the patients had a satisfactory outcome, which we defined as MRD change or elevation ≥2 mm from preoperative MRD1 with an eyelid height asymmetry of <2 mm with the absence of any complications [18]. Only a few studies have been done with such a large follow-up. A similar attrition in MRD1 was seen by Nucci et al. [22] in their retrospective study on 20 infants with severe congenital ptosis followed up for five years. However, they noted the maximum decrease in MRD1 in the first year. In their study, 2/20 needed a re-surgery by the end of five years, whereas in our study, 4/27 patients needed a re-surgery due to recurrence.

The use of a silicone sling has been retrospectively studied by Carter et al. [12] on 61 lids, of which 14 children were under three years of age (mean age: 18 months) with severe ptosis with poor levator function. They reported good to excellent outcomes in all the patients with a mean follow-up of 22 months. They reported a recurrence rate of 7% within 22 months. Landau Prat et al. [23] assessed the outcome of frontalis sling surgery using silicone in 208 patients with simple and complex severe ptosis. They reported a favorable outcome in 70% of their patients. Choi and Kim [24] reported favorable outcomes in almost all their patients having severe ptosis associated with third nerve palsy after a mean follow-up of 32 months. Despite having poor Bell’s scores in most of their patients, they concluded that silicone is a safe material for suspension. Similar results were seen in a retrospective analysis done by Shah and Mukherjee [25] on 25 eyes of 19 patients with ptosis associated with poor Bell’s scores. They concluded that the elastic nature of silicone rods makes it an ideal suspensory material for patients with CPEO or third nerve palsy [25]. A good cosmetic correction was achieved in 34 eyes (89.4%) after a mean follow-up of 18 months (range: 6 to 60 months) in a retrospective study by Bansal and Sharma [26].

Various comparative studies also show favorable results with silicone sling. A comparison between PFL and the frontal sling was done by Hersh et al., and they followed their patients for a mean duration of 46 months [11]. Their success rates were comparable for both groups, with a success rate of 60% in the PFL group and 67.2% in the silicone rod group. Additionally, they found lower rates of ptosis recurrence in the silicone rod group (13%) versus the PFL group (35.3%). Ben Simon et al. studied the efficacy of various sling materials retrospectively in 99 patients and found no statistical difference in the success rate and recurrence of cases done with alloplastic material versus autogenous fascia lata [17].

The reported recurrence rates for silicone rod suspension range from as low as 7% to as high as 44% [11-13,17]. In this study, the recurrence rate was quite low (14.8%). Carter et al. reported a recurrence of 7% (4/61 eyelids) with 22 months of follow-up [12]. Hersh et al. noted a recurrence rate of 13% in their study [11]. Nucci et al. reported a recurrence in two patients in their series of 20 infants [25]. The wide range of reported postoperative ptosis recurrence can be explained by the difference in the follow-up duration in various studies, different sling configurations and a variety of diseases among the patients studied.

Some studies have found that the chances of infection, extrusion, and granuloma formation are higher with synthetic materials [2]. In our study, too much granuloma formation was seen in three patients with tube extrusion seen in two patients. Bansal and Sharma noted complications such as significant eyelid lag and lagophthalmos (five eyes), under-correction (four eyes), suture granuloma (three eyes), sling exposure at forehead incision (three eyes), bilateral chronic eyelid edema (one patient), and late recurrence of ptosis (one eye) [26]. To prevent these complications, a large pocket should be constructed beneath the forehead incision, which should then be meticulously closed in two layers.

The drawback of our study arises from its retrospective design. We did not conduct a formal sample size calculation in this retrospective study. Other limitations include patient selection bias and lack of a control group with other sling materials. Our study was not a comparative analysis of the result of frontalis suspension using different materials. Prospective, randomized, controlled trials should be done using different sling materials to compare the results with different sling materials.

## Conclusions

In conclusion, silicone sling is an effective sling material. Our study establishes that using silicone rods as a sling material provides good cosmesis. The correction persisted through three years of our follow-up with a recurrence rate of only 14% in our study. Satisfactory outcomes were seen in 70% of our patients. The complications seen are few, and it provides good long-term stability. To the best of our knowledge, this is one of the few studies with a follow-up of three years to establish the long-term stability of silicone sling as a sling material. Prospective, randomized controlled trials are required to further elucidate the difference in outcome of ptosis correction using different sling materials.
